# The subjective experience of transcranial electrical stimulation: a within-subject comparison of tolerability and side effects between tDCS, tACS, and otDCS

**DOI:** 10.3389/fnhum.2024.1468538

**Published:** 2024-10-23

**Authors:** Jovana Bjekić, Marko Živanović, Marija Stanković, Dunja Paunović, Uroš Konstantinović, Saša R. Filipović

**Affiliations:** ^1^Human Neuroscience Group, Centre for Neuroscience and Neuromodulation, Institute for Medical Research, University of Belgrade, Belgrade, Serbia; ^2^Institute of Psychology, Department of Psychology, Faculty of Philosophy, University of Belgrade, Belgrade, Serbia

**Keywords:** transcranial electrical stimulation (TES), transcranial direct current stimulation (tDCS), transcranial alternating current stimulation (tACS), oscillatory transcranial direct current stimulation (otDCS), tolerability, side effects, safety, subjective experience

## Abstract

Low-intensity transcranial electrical stimulation (tES), including techniques like transcranial direct current stimulation (tDCS), transcranial alternating current stimulation (tACS), and oscillatory transcranial direct current stimulation (otDCS), has been widely explored for its neuromodulatory effects on motor, cognitive, and behavioral processes. Despite well-established safety, these techniques can induce varying degrees of discomfort and side effects, potentially impacting their application. This study presents a within-subject sham-controlled experiment directly comparing the subjective experience and side effects of tDCS, tACS, and otDCS. Participants reported their discomfort levels at multiple time points during 20-min stimulation sessions and completed a side-effects questionnaire before and after each session. Results indicated that the overall discomfort levels were low across all conditions, with ≥95% reporting the absence of discomfort or mild procedure-induced discomfort. Nevertheless, tDCS and otDCS were slightly less comfortable compared to sham, especially at the beginning of stimulation, with tACS-induced discomfort levels being overall comparable to sham. The most common side / adverse effects were mild skin sensations, including itching and tingling, particularly with tDCS and otDCS, while tACS occasionally caused phosphenes and blurred vision. These findings provide a systematic comparison of tES-induced discomfort and side effects between different tES techniques, highlighting the high safety of tES, but also the importance of considering within- and between-person variability and time-course effects in tES applications.

## Introduction

Low-intensity transcranial electrical stimulation (tES) encompasses a set of techniques that use weak electrical currents (up to 4 mA) to modulate the activity of neural circuits thus eliciting sensory, emotional, cognitive, and behavioral changes (Antal et al., 2017). These techniques have gained significant interest in the past 15 years in basic cognitive and affective neuroscience research as well as clinical research and practice. The most widely used tES technique is transcranial direct current stimulation (tDCS), which delivers a constant unidirectional current during a certain period of time – usually 10–20 min (Nitsche et al., 2008). In contrast, in transcranial alternating current stimulation (tACS), the current rhythmically switches polarity by following a sinusoidal oscillating waveform at a set frequency (e.g., oscillating at 10 Hz between −1 and +1 mA) (Antal and Paulus, 2013). Finally, the current can be delivered in an oscillatory manner but remaining within the same polarity –in the technique called oscillatory transcranial direct current stimulation (otDCS) the current oscillates at a certain frequency solely within the positive or negative polarity (e.g., between 1 and 2 mA) (Vulić et al., 2021). Differences in the current waveform are responsible for differential neuromodulatory mechanisms of action but can also lead to different sensory perceptions, subjective experiences, and the tolerability of the procedure (Fertonani et al., 2015).

Even though the safety of low-intensity tES, especially conventional tDCS has been firmly established (Antal et al., 2017), mild to moderate side effects usually in a form of unpleasant cutaneous sensations or transitory skin reactions beneath the electrodes are quite common. This was well documented by several large-scale studies focusing on the side effects of tDCS (see Brunoni et al., 2011; Kessler et al., 2012; Nitsche et al., 2003; Poreisz et al., 2007; Russo et al., 2013). However, these studies indicated that tDCS-related side effects are not only sensory, but may include difficulties concentrating, tiredness, headaches, nausea, and a general sense of discomfort. Interestingly, most of the side effects recorded during or shortly after tDCS were also reported when sham protocols were applied (Brunoni et al., 2011; Poreisz et al., 2007) suggesting a possibility of placebo/nocebo effects.

The subjective experience and side effects differ between techniques and may depend on stimulation parameters such as intensity of the current, current waveform as well as position and size of the electrodes. Namely, it was shown that higher intensities and larger electrodes induce stronger sensations (Fertonani et al., 2015; Turi et al., 2014), while techniques with oscillatory currents produce sensations of comparatively less intensity (Fertonani et al., 2015). Still, the oscillations may lead to other phenomena such as flickering or shaking of the visual field and phosphenes, which are not observed in tDCS (Antal et al., 2017; Lang et al., 2019).

Here, we comparatively assessed the subjective experience, i.e., procedure-induced discomfort and potential side effects of three types of tES - anodal tDCS, tACS, and otDCS in a same group of participants, to provide a systematic and comprehensive comparison between these tES techniques. Even though reports on side effects and tolerability of oscillatory tES protocols can be found in some studies (see e.g., Lang et al., 2019; Vulić et al., 2021; Živanović et al., 2022) evidence on a direct comparison between different tES techniques is still limited. Furthermore, a careful review of the data presented in the literature shows quite variable incidence of each side effect, thus highlighting the variability in subjective experience across participants. Namely, looking at the tES-induced sensations and side effects from different groups is by design confounded by the individual differences between participants in terms of their sensitivity, tolerability, and expectations. Thus, only data from the same group of participants provides a reliable basis for comparison between different tES techniques. Furthermore, while there is an abundance of data regarding tDCS, the data on tACS-induced side effects are less systematic, while the reports on the subjective experience of otDCS are almost completely lacking. Finally, to the best of our knowledge, there is no data showing the tES-induced discomfort at different time-points during the stimulation as well as pre-to-post differences between these techniques.

## Methods

### Design

We conducted a within-subject sham-controlled experiment in which participants received tDCS, tACS, otDCS or sham stimulation in four sessions at least 7 days apart. The stimulation conditions were delivered in counterbalanced order, and subjective experience was assessed before, during, and after the stimulation. The data presented here are part of the MEMORYST project (for study protocol see Bjekić et al., 2022b). The experiment was conducted in line with the guidelines of the Declaration of Helsinki and was approved by the Institutional Ethics Board (EO129/2020).

### Participants

A group of 42 young adults (age: 22 – 34 years, *M* = 25.05, SD = 3.55; 16 male and 26 female) took part in the study. All participants were without history of psychiatric or neurological disorders, naïve to tES, right-handed, with normal or corrected-to-normal vision, and satisfied common low intensity tES inclusion criteria (Antal et al., 2017). All participants gave their written informed consent prior to the experiment and received financial compensation for their participation.

### Low intensity tES

**FIGURE 1:**
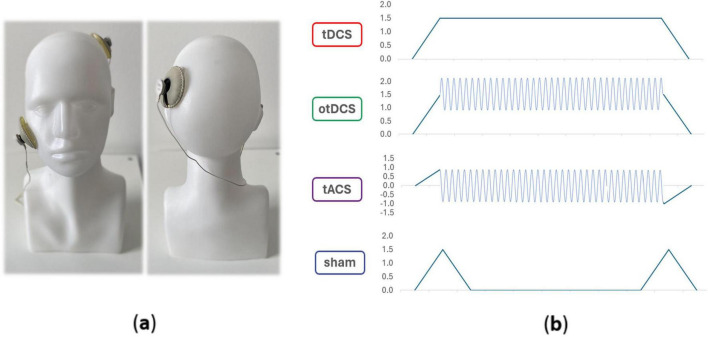
Electrode position **(a)** and schematic presentation of tES waveforms in different conditions **(b)**.

The tES was administered using the Starstim32 device (Neuroelectrics Inc., Barcelona, Spain), remotely operated through the Neuroelectrics^®^ Instrument Controller (NIC2) software (Neuroelectrics Inc., Barcelona, Spain). We used 1 × 1 electrode set-up (Figure 1) – one electrode was placed over the left parietal cortex (P3 according to the international 10-10 EEG positioning system) and secured with the Neoprene Headcap; the second electrode was secured with medical adhesive tape on the contralateral cheek. Round rubber electrodes (25 cm^2^) inserted in saline-soaked sponge pockets were used. Each participant underwent four stimulation conditions: tDCS, otDCS, tACS, and sham (Figure 1). The intensity of constant anodal tDCS was 1.5 mA. tACS was delivered as a sinewave of 0±1 mA (2 mA peak-to-peak) at theta frequency (4–8 Hz). The frequency was individually determined for each participant (Bjekić et al., 2022a). The otDCS was also delivered at the same theta frequency, with the current oscillating between 1 and 2 mA (1.5 ± 0.5 mA). In all active tES conditions, the stimulation was delivered for 20 min with 30 s gradual ramp-up at the beginning and 30 s ramp-down at the end. In sham condition, the current was gradually ramped up to 1.5 mA (30 s) and down to 0 mA (30 s) at the beginning and at the end of stimulation period.

### Assessment of tES-induced discomfort

The subjective experience, i.e., induced discomfort during the stimulation was measured using a 10-point scale (1 – absence of any discomfort, 10 – extreme discomfort). The discomfort ratings were collected at predefined time points (minutes 1, 8, 16, and 20 of the stimulation) and were prompted by the experimenter asking: “Please tell me how you feel right now on a scale from 1 meaning *I do not feel any discomfort* to 10 meaning *I feel extreme discomfort*”.

### Measures of potential side-effects

The potential side-effects were assessed by Symptoms self-report questionnaire (adapted from Antal et al., 2017) and filled out by participants before and immediately after each stimulation session. Participants were alone in the testing boot, to avoid distractions or experimenter effects (e.g., social desirability bias) on the reported sensations. The questionnaire consisted of 13 potential side-effects: headache, neck pain, back pain, blurred vision, skin irritation, prickling/tingling sensation, itching, increased heart rate, burning sensation, dizziness, acute mood swings, tiredness, anxiety. Participants were asked to rate each on a 10-point scale (“To what extent are you experiencing each of the following”; 1 – not at all, 10 – extremely). At the end of the questionnaire, participants were asked to report on any symptoms or side-effects not already listed. Spontaneous comments made by the participants during or following the stimulation of any adverse effects were recorded by the researcher.

### Blinding assessment

The successfulness of participants-blinding was assessed by end-of-study guess of sham condition. At the end of the last stimulation session, participants were reminded of the types of stimulation they had received and were asked to guess the session in which they believed they had received sham stimulation.

### Data analysis

The comparisons between different tES conditions were made in the series of repeated measures ANOVAs with factors STIMULATION CONDITION (4 levels: tACS, otDCS, tDCS and sham) and TIME POINT (minute 1, 8, 16, and 20) with reported level of discomfort as dependent variable. The ratings of discomfort (10-point scale) were interpreted as: ≤2 absence of discomfort; 2 < x ≤ 5 mild discomfort; 5 < x ≤ 8 high discomfort; >8 extreme discomfort. For each of the potential side effects, the baseline-to-post stimulation changes were assessed using the paired *t*-tests. The stimulation conditions were compared in the repeated measures ANOVAs with difference score (post stimulation – baseline rating) for each symptom as a dependent variable. The procedure-induced side effects were interpreted as: baseline-to-post stimulation difference 0 – no side effects, 1-2 negligible side effects, 2-3 mild side-effects induced, >3 noticeable side effects. For each ANOVA model the partial eta squared (η_p_^2^) was used as the effect-size estimate. Bonferroni-corrected *post hoc* tests were used for comparisons between stimulation conditions. The successfulness of blinding was assessed using the one-tailed Binomial test with pre-defined probabilities, i.e., for sham guess the alternative hypothesis (H1) was *p* > 0.25. Finally, we assessed the relationship between reported discomfort in each stimulation condition and sham-guess (0/1) using the point-biserial correlation coefficients.

## Results

### tES induced (dis)comfort

**FIGURE 2:**
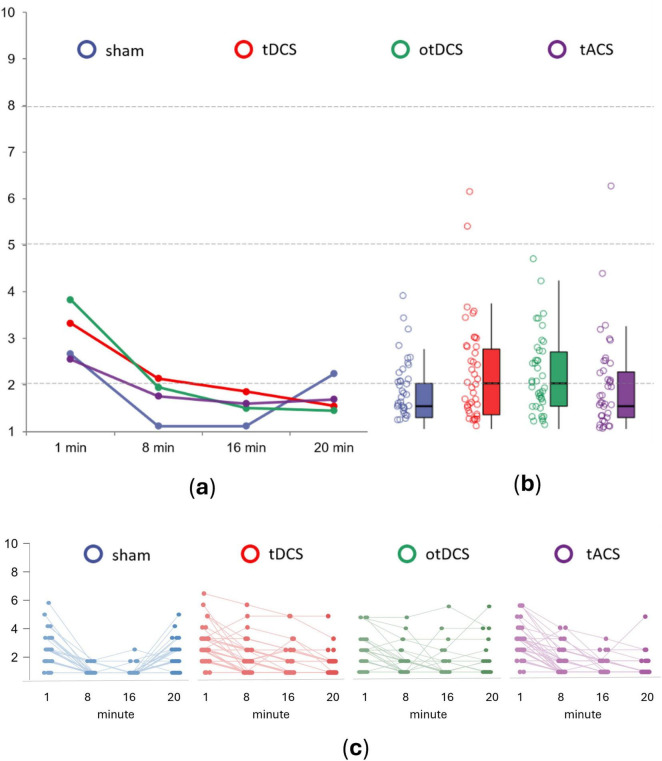
The average procedure induced discomfort for sham (blue), anodal tDCS (red), otDCS (green), and tACS (purple) at panel **(a)** different time points of stimulation – minute 1, 8, 16 and 20, **(b)** when aggregated across all time-points, and **(c)** at the individual level. The values ≤2 indicate absence of discomfort; values 2 < x ≤ 5 indicate mild discomfort; values 5 < x ≤ 8 indicate high discomfort; and values >8 indicate extreme discomfort.

The analysis at the individual level showed that almost all participants reported either the absence of discomfort or mild procedure-induced discomfort (Figure 2). Specifically, the absence of discomfort was reported by the majority of participants across all stimulation conditions (tDCS 57.1%, otDCS 57.1%, tACS 71.4%, and sham 78.6%). Mild discomfort was reported by 38.1% of participants during tDCS, 42.9% during otDCS and 26.2% during tACS, while 21.4% of participants had mild discomfort during sham. High levels of discomfort were reported only by two participants during tDCS and one participant during tACS, while none reported extreme discomfort in any stimulation condition.

At the group-level, procedure-induced overall discomfort was low across all tES conditions [tDCS (*M* = 2.22, SD = 1.14), otDCS (*M* = 2.18, SD = 0.87), and tACS (*M* = 1.89, SD = 1.02)], and similar to the sham (*M* = 1.74, SD = 0.66). Still, repeated measures 4 × 4 ANOVA showed the main effect of STIMULATION [*F*_(3,123)_ = 4.170, *p* = 0.008, η_p_^2^ = 0.092]. Specifically, in comparison to sham, tDCS and otDCS were slightly more unpleasant (*p* = 0.015 and *p* = 0.003, respectively), while tACS induced same levels of (dis)comfort as sham (*p* = 1.000). However, no significant differences between different tES techniques were recorded (tDCS *vs* otDCS *p* = 1.000, tDCS *vs* tACS *p* = 0.769, otDCS *vs* tACS *p* = 0.488).

In addition, we observed a large main effect of TIME (*F*_(3,123)_ = 87.677, *p* < 0.001, η_p_^2^ = 0.681), as well as STIMULATION x TIME interaction (*F*_(9,369)_ = 10.357, *p* < 0.001, η_p_^2^ = 0.202). The highest levels of discomfort were reported in the first minute (all *p* < 0.01), gradually decreasing as the stimulation progressed (8th min *vs* 16th min *p* = 0.002) before finally reaching a plateau by the end of stimulation (16th min *vs* 20th min *p* = 0.056) (Figure 2A). Hence, the largest differences between tES conditions were found at the beginning of stimulation (*F*_(3,123)_ = 11.503, *p* < 0.001, η_p_^2^ = 0.219), where sham induced lower levels of discomfort than tDCS (*p* = 0.043) and otDCS (*p* = 0.001), but not tACS (*p* = 1.000). Here, tDCS induced a similar level of discomfort as otDCS (*p* = 0.155), and both were rated as more unpleasant than tACS (tDCS *vs* tACS *p* = 0.032 and otDCS *vs* tACS *p* < 0.001).

Similarly, after 8 min, there was a significant effect of STIMULATION (*F*_(3,123)_ = 8.709, *p* < 0.001, η_p_^2^ = 0.175), with all active tES conditions being experienced as less pleasant than sham (tDCS *vs* sham *p* < 0.001, otDCS *vs* sham *p* < 0.001, and tACS *vs* sham *p* = 0.005). In the second half of the stimulation period (min 16), the difference between tES conditions still remained statistically significant (*F*_(3,123)_ = 4.494, *p* = 0.005, η_p_^2^ = 0.099). However, the difference between sham and active tES conditions became smaller (tDCS *vs* sham *p* = 0.004, otDCS *vs* sham *p* = 0.006) with tACS *vs* sham difference disappearing (*p* = 0.109). Interestingly, after habituation (min 8 and 16) the differences between tACS, otDCS, and anodal tDCS were no longer observed (all *p* > 0.250).

Again, at the end of the stimulation period, the main effect of STIMULATION was found (*F*_(3,123)_ = 2.876, *p* = 0.039, η_p_^2^ = 0.066), with sham being experienced as marginally less pleasant than otDCS (*p* = 0.054), while the same trend was observed for tDCS (*p* = 0.087) but not tACS (*p* = 0.693). Finally, no differences between active tES conditions were observed at the last time-point (all *p* = 1.000).

### Potential side effects

Overall, ratings across all recorded symptoms, both pre- and post-stimulation, were relatively low across all tES conditions, with the average ratings < 2 for all (except for the tiredness, see Table 1). The main effect of the STIMULATION was observed only for scalp irritation, while post-hoc comparisons between tES conditions were not statistically significant for any of the potential side effects (all *ps* > 0.05).

**TABLE 1:** The reported severity of side-effects at baseline (pre-stimulation) and post-stimulation, with pre- to post-stimulation comparisons (*t*-tests) within each condition separately (sham, tDCS, otDCS, tACS), and repeated measure ANOVAs testing the differences between baseline-to-post-stimulation changes in symptoms’ severity between stimulation conditions.

Symptom	Sham^1^			tDCS^1^			otDCS^1^			tACS^1^			Main effect^4^		
	pre^2^	post	*t*-test^3^	pre	post	*t*-test	pre	post	*t*-test	pre	post	*t*-test	*F*	*p*	η _p_^2^
Headache	1.31	1.48	0.943	1.50	1.64	1.030	1.38	1.45	0.829	1.43	1.69	1.808	0.402	0.752	0.010
	(0.68)	(1.23)		(0.99)	(1.21)		(0.79)	(0.74)		(1.17)	(1.44)				
Neck pain	1.21	1.29	1.355	1.36	1.36	0.000	1.31	1.40	1.432	1.45	1.45	0.000	0.274	0.844	0.007
	(0.47)	(0.60)		(0.88)	(0.66)		(0.60)	(0.77)		(0.86)	(0.71)				
Back pain	1.55	1.55	0.000	1.55	1.52	0.443	1.50	1.45	0.703	1.52	1.48	0.703	0.133	0.940	0.003
	(1.06)	(1.13)		(1.06)	(1.06)		(0.83)	(0.74)		(0.89)	(0.77)				
Blurred vision	1.33	1.43	1.432	1.29	1.36	1.138	1.24	1.31	1.355	1.24	1.48	2.125*	2.050	0.110	0.048
	(0.90)	(0.97)		(0.89)	(0.93)		(0.85)	(0.87)		(0.85)	(1.02)				
Scalp irritation	1.19	1.31	1.044	1.12	1.62	2.713*	1.05	1.26	2.672*	1.07	1.17	1.667	3.250	**0.024**	0.073
	(0.55)	(0.78)		(0.40)	(1.19)		(0.22)	(0.59)		(0.26)	(0.44)				
Tingling sensation	1.02	1.38	3.048**	1.05	1.71	3.939**	1.02	1.45	3.232**	1.02	1.26	1.880	2.306	0.080	0.053
	(0.15)	(0.76)		(0.31)	(1.07)		(0.15)	(0.86)		(0.15)	(0.83)				
Itching	1.21	1.45	2.354*	1.21	1.76	2.924**	1.14	1.52	2.386*	1.14	1.26	1.403	2.190	0.093	0.051
	(0.52)	(0.86)		(0.61)	(1.41)		(0.52)	(1.19)		(0.42)	(0.50)				
Increased heart rate	1.29	1.19	1.432	1.29	1.14	1.961	1.29	1.14	1.635	1.17	1.14	0.374	0.598	0.618	0.014
	(0.89)	(0.97)		(0.77)	(0.47)		(0.89)	(0.47)		(0.44)	(0.65)				
Burning sensation	1.05	1.10	0.813	1.07	1.26	1.598	1.00	1.19	2.442*	1.02	1.19	1.361	0.529	0.663	0.013
	(0.22)	(0.37)		(0.34)	(0.73)		(0.00)	(0.51)		(0.15)	(0.94)				
Dizziness	1.10	1.17	0.723	1.14	1.14	0.000	1.12	1.14	0.374	1.12	1.24	1.403	0.658	0.580	0.016
	(0.37)	(0.49)		(0.47)	(0.52)		(0.40)	(0.47)		(0.33)	(0.66)				
Acute mood swings	1.19	1.21	0.227	1.24	1.14	1.432	1.19	1.12	1.138	1.19	1.19	0.000	0.768	0.514	0.018
	(0.40)	(0.81)		(0.73)	(0.42)		(0.45)	(0.40)		(0.51)	(0.51)				
Tiredness	2.21	2.57	1.704	2.21	2.48	1.426	1.81	2.00	2.077*	2.31	2.40	0.644	0.841	0.474	0.020
	(1.20)	(1.76)		(1.41)	(1.86)		(1.04)	(1.19)		(1.51)	(1.65)				
Anxiety	1.52	1.33	2.238*	1.67	1.40	2.127*	1.55	1.26	2.751**	1.52	1.57	0.404	2.532	0.060	0.058
	(1.17)	(0.82)		(1.37)	(0.89)		(1.06)	(0.54)		(0.94)	(0.99)				

^1^sham; tDCS – constant anodal transcranial direct current stimulation; otDCS – oscillatory transcranial direct current stimulation; tACS – transcranial alternating stimulation;

^2^pre- and post-stimulation ratings – mean and standard deviation is presented as *M*(*SD*);

^3^The absolute value of t-statistic,

*p < 0.05;

**p < 0.01;

^4^The repeated measures ANOVA with factor STIMULATION CONDITION – *F*-statistic, exact *p-*value and the measure of the effect size (η_p_^2^). Bold are significant values (either p or f).

**FIGURE 3:**
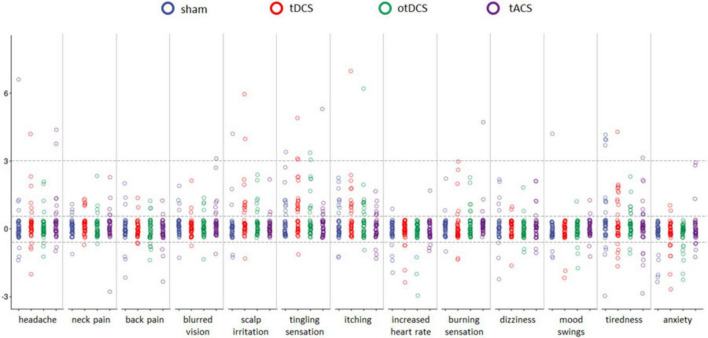
The pre-stimulation to post-stimulation differences (*y*-axis) for each of 13 potential side effects (*x*-axis) for sham (blue), anodal tDCS (red), otDCS (green), and tACS (purple). The value 0 marks the absence of procedure-induced side effects (i.e., no baseline-to-post stimulation change); positive values indicate an increase in symptom intensity following the stimulation (≤3 negligible-to-mild, ≥3 notable side-effects), while negative values indicate a decrease in intensity of a symptom in comparison to pre-stimulation baseline. Individual data points are jittered for visualization purposes i.e., all data points between the dotted lines around 0 have value 0.

Even though baseline-to-post stimulation changes in symptoms’ intensity were small, statistically significant changes were observed across some of the symptoms (Table 1). As shown in Figure 3, some participants showed changes post-stimulation relative to baseline. Namely, following the tDCS and otDCS, significant changes in several skin sensations were reported – 13 participants (31.0%) post-tDCS and 11 participants (26.2%) post-otDCS reported increased scalp itching; 18 participants (42.9%) noted increased tingling sensation post-tDCS and 10 participants (23.8%) post-otDCS; 12 participants (28.6%) and 7 participants (16.7%) post-otDCS reported negligible-to mild scalp irritation post-tDCS, while negligible-to-mild burning sensation was reported by 6 participants (14.3%) following otDCS. It is important to note that similar skin-sensation aftereffects were recorded following sham stimulation too. In addition to that, 7 participants (16.7%) reported negligible-to-mild blurred vision following tACS and 8 participants (19.1%) reported increased tiredness following otDCS.

In addition to the listed symptoms, phosphenes were reported by three participants during tACS and one participant during otDCS, while one participant reported shaking of the visual field at the beginning of tACS. Two participants had right-side facial muscle twitching during tACS. Finally, a metallic taste was reported by two participants during tACS and one during tDCS and otDCS, each.

Interestingly, some positive side effects of tES were observed as well. Namely, the reported levels of anxiety were lower following stimulation than at baseline in both tDCS and otDCS (8 participants, i.e., 19.1% each) as well as sham condition (5 participants, i.e., 11.9%). At the level of individual participants, beneficial effects of stimulation were recorded for other symptoms too (Figure 3), but these effects were not systematically observed at the group level.

### Effects on blinding

The one-tailed Binomial test showed that the sham-guess proportion (0.262) was not greater than chance-level (*p* = 0.488). tACS, tDCS, and otDCS were mistaken for sham in 33.3% of cases, 23.8 and 16.7%, respectively. Notably, the successful sham-guess was not related to the reported discomfort in any of the stimulation conditions (sham: *r* = −0.202, *p* = 0.199; tDCS: *r* = −0.044, *p* = 0.781; otDCS: *r* = 0.076, *p* = 0.630; tACS *r* = 0.134, *p* = 0.398).

## Discussion

This study provides a systematic and comprehensive comparison of the subjective experiences, including procedure-induced discomfort and potential side effects, associated with three types of tES: anodal tDCS, tACS and otDCS. By evaluating these effects within the same group of participants, we sought to address the variability in subjective experiences across different tES techniques and account for individual differences that often confound cross-study and between-groups comparisons.

Our findings indicate that the overall procedure-induced discomfort was low across all tES conditions including the sham (with absence or mild discomfort reported by >95% of participants regardless of the type of stimulation), suggesting that none of the techniques were uncomfortable for participants. Nevertheless, we noted statistically significant overall differences between stimulation conditions. Specifically, both tDCS and otDCS were perceived as slightly more unpleasant compared to sham, with no differences between them, which could be expected due to the equal current intensities of the two (1.5 and 1.5 mA ± 0.5 mA). Similar to previous reports (Fertonani et al., 2015), tACS-induced discomfort was no different than sham. However, this may be due to the fact that the maximal amplitude of tACS in either polarity did not exceed 1 mV and thus was smaller than the anodal amplitude in both tDCS and otDCS suggesting that the discomfort level likely depends more on the maximal intensity of the stimulation rather than the absolute amplitude of the oscillations.

Overall, our data aligns with previous reports suggesting variability in tolerability among different tES procedures (Sheffield et al., 2022) and underscores the importance of considering individual stimulation modalities separately when evaluating patient/participant comfort. Here it is important to highlight that at the individual level, the majority of participants reported an absence of discomfort across all stimulation conditions. When discomfort was present, it was generally mild, with only a small number of participants experiencing higher levels of discomfort. This pattern is consistent with the tolerability profiles reported in previous studies (Matsumoto and Ugawa, 2017), reinforcing the notion that major discomfort is rare during tES. Furthermore, the successful participants blinding was shown, as correct sham guess was no different than chance level and was not related to the reported discomfort levels. Participants unblinding is especially at risk in within-subject designs, as repeated exposure to stimulation may lead to increased awareness based on the experience with different stimulation protocols. However, it seems that overall high tolerability and minimal side effects ensured that the majority of participants remained unaware of the stimulation condition. This is especially the case for tACS, which was more often mistaken for sham than otDCS or tDCS.

The time course analysis revealed a dynamic pattern of discomfort over the stimulation period. In line with other studies (Wallace et al., 2016), the reported discomfort was highest in the first minute, with significant differences between tES conditions during this initial phase. Notably, both tDCS and otDCS were rated as more unpleasant than tACS at the beginning of the stimulation, highlighting potential differences in the onset sensations induced by these techniques. Over time, however, the level of stimulation-induced discomfort gradually decreased and differences between active tES conditions diminish, particularly after the 8-min mark, suggesting habituation effects. By the end of the stimulation period, no significant differences between the active tES conditions were observed, indicating that initial discomfort levels might not predict the overall tolerability of the procedure. Additionally, our data imply sensitivity to the change in current intensity, which is best illustrated by the increased discomfort reported during ramp-up/down at the end of the sham protocol.

The findings have important implications for the design of future studies assessing behavioral effects (motor or cognitive) during tES (so-called online protocol). Namely, since procedure-induced discomfort may influence the performance, subjective experience with its temporal dynamic may act as a confounding factor for the behavioral effects. Therefore, it is advisable to (1) record discomfort levels at multiple time points during the stimulation and analyze them alongside behavioral performance, and (2) start with behavioral tasks a few minutes (e.g., 3–5 min) after the onset of stimulation when the initial intensity of sensations has decreased, and (3) assess sham-guessing to ensure that even these minimal sensations did not compromise the blinding of participants.

In terms of side / adverse effects, our data showed low average ratings across all recorded symptoms pre- and post-stimulation, with significant changes observed primarily for scalp sensations. Namely, the most pronounced side effects found overall were mild cutaneous sensations of tingling and itching under or close to the electrodes. These adverse effects were observed following tDCS and otDCS but were also noted to some extent after sham stimulation. This finding validates the effectiveness of the double ramp up/down sham procedure as it produces similar skin-sensations as tDCS/otDCS and therefore adds to masking for the type of stimulation they are receiving. The occurrence of elevated cutaneous sensations is in line with the results of the systematic review of 209 studies that found tingling and itching to be the most commonly reported tDCS-induced side-effects (Brunoni et al., 2011). Our study, as well as most of the previous reports, showed minimal adverse effects following one tDCS session, but a recent study also reported similar side effects in multiple tDCS sessions (Delicado-Miralles et al., 2024). This further supports the notion that only low to mild adverse effects can occur after a single as well as after repeated exposure to tDCS.

Interestingly, although typically reported for tDCS, we recorded no tACS-induced skin sensations. These side effects are also less commonly reported for tACS in the literature. One possible reason is that tACS does not create long-term polarization of the tissue (Fertonani et al., 2015). Conversely, tACS was associated with unique side effects, which are consistent with the sensory phenomena previously reported in the literature (Antal et al., 2017; Elyamany et al., 2021; Matsumoto and Ugawa, 2017). We recorded three cases of tACS-induced phosphenes, and several participants reported blurred vision following this stimulation protocol. These effects could probably be attributed to the position of the reference electrode on the contralateral cheek, as electrical field modeling studies have provided evidence that the tACS-induced flickering was of retinal rather than cortical origin (Laakso and Hirata, 2013), and that the stronger phosphenes were elicited when at least one of the electrodes is placed in proximity to the eye (Kanai et al., 2008; Schutter and Hortensius, 2010; Turi et al., 2014). In contrast to some previous findings demonstrating that tACS with posterior montages induces dizziness at slow theta frequencies (4 Hz) (Raco et al., 2014), we did not record this side effect for theta-tACS or theta otDCS. On the other hand, increased tiredness was reported following otDCS, but not tDCS and tACS. However, in the absence of at least trend-level differences between otDCS and other stimulation conditions, we cannot be certain that this side-effect is exclusively induced by otDCS. This might well be simply a chance-finding since we found no previous reports of such an effect. Nevertheless, it is important to highlight that in this study all oscillatory tES were delivered in theta-band (4–8 Hz), and that the tES-induced sensations might differ in intensity and frequency of occurrence if other frequencies were used. This frequency dependence was shown previously by Turi et al., 2013 who explored cutaneous sensations and phosphenes for a wide range of tACS frequencies (2–250 Hz), and found that the strongest sensations were perceived in the beta and gamma frequency range, especially at 20 Hz, while the ripple range frequencies (140 and 250 Hz) were almost nondetectable.

It should be noted that some of the participants reported symptoms that might be considered as potential stimulation side effects (e.g., headache, itching, tiredness) even before any stimulation was delivered. This implies that looking only at post-stimulation reports might lead to false attribution of all elevated symptoms to the stimulation. Furthermore, the role of non-stimulation-related factors in the generation of the reported symptoms can be strongly suspected when these symptoms are already present before stimulation, thus one should be careful in attributing changes in the level of these symptoms solely to the neurobiological effects of the stimulation. This underlies the importance of systematic recording of all symptoms considered as potential side effects both before and after the stimulation and assessing their significance in individualized manner.

The occurrence of positive side effects, such as reduced anxiety post-stimulation, was an unexpected finding. This effect was observed across tDCS, otDCS, and sham conditions, suggesting a potential placebo effect or an underlying psychophysiological response to the stimulation setup. In other words, it is likely that the participants encountering tES for the first time, have different expectations and that the anticipation of the new experience causes elevated levels of anxiety, which disappears as they complete the stimulation. This is also an important factor to consider when evaluating the behavioral effects of tES, as pre-stimulation anxiety may act as a distractor and modulate attention. Notable, the other “positive” effects were recorded too (e.g., lower reports of headache or increased heart rate and tiredness), but they were not systematically observed at the group level, which highlights the complexity of tES effects on subjective experience and warrants further investigation.

Finally, it is important to draw attention to the inter-individual variability across all recorded measures including both measures of (dis)comfort during tES as well as post-stimulation side effects. Therefore, it is important that the stimulation procedures and protocols are set up with the awareness of the participants’ pre-stimulation state, as well as the possibility that some people may be highly sensitive and experience elevated levels of discomfort, during otherwise safe and well-tolerated protocols. This is essential for ensuring adherence to the ethical implementation of these techniques.

## Conclusion

In summary, our study provides valuable insights into the subjective experiences associated with different tES techniques. The data presented here add to the body of evidence supporting the safety and tolerability of these techniques. While all three tES modalities were generally well-tolerated, there were notable differences in the onset and temporal dynamic of discomfort, as well as in the specific side effects associated with each technique, and a high level of between-person variability. These findings underscore the importance of considering individual tES techniques separately when assessing their tolerability and side effects and provide a foundation for future research aimed at optimizing tES protocols for clinical and research applications.

## Data Availability

The datasets presented in this study can be found in online repositories. The names of the repository/repositories and accession number(s) can be found below: https://osf.io/tj2ed/.
